# A Three-Fold Integrated Perspective on Healthy Development: An Opinion Paper

**DOI:** 10.3390/brainsci13060857

**Published:** 2023-05-25

**Authors:** Patrizio Paoletti, Michele Pellegrino, Tal Dotan Ben-Soussan

**Affiliations:** Research Institute for Neuroscience, Education and Didactics, Patrizio Paoletti Foundation for Development and Communication, 06081 Assisi, Italym.pellegrino@fondazionepatriziopaoletti.org (M.P.)

**Keywords:** mental health, self-awareness, emotional intelligence, wellbeing, EEG, Quadrato Motor Training, pro-NGF, pro-BDNF, neuroeducation

## Abstract

Mental health and wellbeing are increasingly threatened in the current post-pandemic times, with stress, especially in students, reaching preoccupying levels. In addition, while many educational programs are unidimensional (i.e., lacking integration between physical, emotional and cognitive elements), there are ways to promote physical, social and mental health in children and adolescents. In this opinion paper, we will discuss the importance of an integrative approach for health development and examine relevant factors, such as awareness and emotional intelligence. We will highlight evidence ranging from behavioral to electrophysiological, structural and molecular, and report several recent studies supporting the effectiveness of a holistic approach in supporting wellbeing and creativity in children and adults, and detailing a specific paradigm named the Quadrato Motor Training (QMT). QMT is a specifically structured movement meditation, involving cognitive, motor and affective components. Finally, we will support a holistic view on education, integrating motion, emotion and cognition to develop a person-centered, or in this case student-centered, approach to wellbeing and health.

## 1. The Importance of Emotional Wellbeing in Post-Pandemic Times

The Global Burden of Diseases, Injuries and Risk Factors Study (GBD) reported that the two most disabling and burdening mental disorders before the pandemic were anxiety and depression [1]. The impact of these mental disorders was across the entire lifespan, for both sexes and worldwide [1]. These data were from a pre-pandemic period. The COVID-19 pandemic in 2020 and the following years, in addition to the health risks involved, has given rise in the prevalence of stress which has led to increased depressive and anxiety disorders by over than 25% [2,3]. Sadly, despite different attempts to promote mental health, the pandemic has especially impacted adolescents and young adults, doubling the prevalence of depressive and anxiety symptoms among youths [4]. Of particular importance, UNESCO, in 2021, declared the COVID-19 pandemic to be the most severe disruption to global education in history, estimating that 1.6 billion learners in over 190 countries have been fully or partially out of school in 2020 [5]. Moreover, not only have learning opportunities been affected, but, with school closures and wider social restrictions in place, students have been unable to meet each other in person, deeply affecting their ability to learn and opportunities for peer interaction [2]. Despite this critical situation, less than a quarter of students reported that they would seek treatment if they had a future emotional problem [6].

Due to the stigma of mental illness and attitudinal barriers to seeking mental health treatment in students, an alternative way of offering help to young people is needed. One way to do so is through stress management interventions [7]. This type of intervention may have two important benefits for reducing the burden of mental illness students. Firstly, as highlighted by Amanvermez and colleagues [8], stress management interventions can also reduce depression, albeit to a lesser extent than they reduce stress and anxiety. Secondly, offering stress management interventions may be a more palatable and attractive way to encourage college students to seek treatment compared to mental health services [7].

Thus, it seems that this type of intervention is not enough. Although physical activity is crucial for cognitive development and emotional wellbeing [9,10,11,12], negative physical health trends in youth are alarming. Around 25% of children and youth do not engage in adequate health-enhancing daily physical activity levels [13], with only 23% of male and 10% of female youth meeting aerobic endurance and muscle strengthening guidelines [14]. Recent data also indicate a large percentage of children are not developing foundational motor skills [9,15]. As variables pertaining to physical health (e.g., time spent doing physical activity, physical fitness, motor development, weight status) are inextricably linked with psychological, social, emotional, behavioral and cognitive development [9,10,11,12], a more integrated way to enhance wellbeing and offer tools of coping with stress, mental and physical health in young students should offer an integrated approach to learning, taking into account multiple domains of health and well-being. This is of utmost importance, especially as children are largely sedentary and do not meet physical activity guidelines. The school setting is where children spend most of their waking hours, with children spending approximately 70% of class time being sedentary [16].

In addition, educational settings, as well as parents and society in general tend to overemphasize the importance of cognitive factors in academic achievements, often at the expenses of promoting other important factors, such as physical activity and social-emotional processes (as claimed, for example, by [17,18], respectively). However, evidence shows that non-cognitive variables, such as emotion and motivation, may predict academic performance (e.g., [19]) and may even better predict academic performance than cognitive factors such as intelligence [20,21]. Yet, while some have emphasized the importance of cognitive–motor interactions (e.g., demonstrating shared functional developmental and evolutionary history [22]), others have further highlighted the connection between motion, emotion and motivation [23]; interventional studies and educational programs do not address these three aspects of cognition, emotion and motion together. In recent years, most educational programs could only be considered as unidimensional from a developmental domain-specific approach, which could severely limit the potential collective outcomes that could be supported by a more integrated approach [24,25,26]. For this reason, in this opinion paper, we aim to emphasize the need to address the physical and the emotional wellbeing of the child in the educative process, which should not only focus on academic grades and competition, but on emotional intelligence and well-being.

Indeed, current research considers emotional intelligence as a protective factor against the adverse effects of psychosocial risks [27]. Emotional intelligence can be defined as the ability to recognize our own and others’ feelings and to motivate and handle our emotions [28]. Evidence suggests that people with higher levels of emotional intelligence tend to be more flexible and recover faster from acute stress [29,30]. As Schneider and colleagues [31] also highlighted, emotional intelligence can facilitate stress responses in the direction of challenge, rather than threat, as a coping attitude. Emotional intelligence was linked to lower threat appraisals, reduced declines in positive affect and less negative affect [31]. The ability to adaptively cope with adversity is further often associated with the construct of resilience [32].

**Figure 1:**
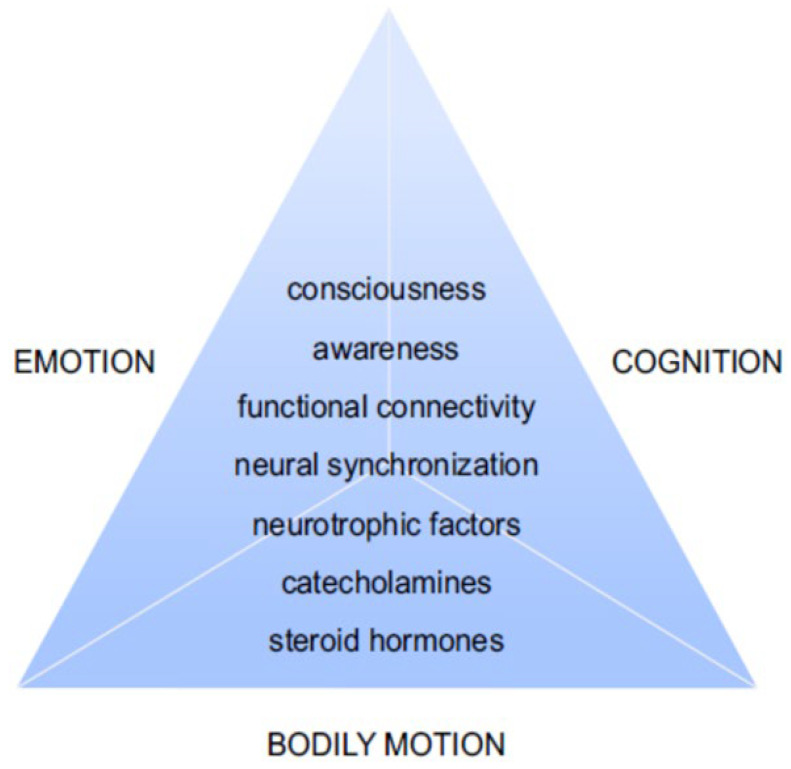
The interconnectedness between motion, emotion and cognition and the possible dimensions that need to be examined in order to achieve a comprehensive overview of the effects of training for healthy development. Adapted from [24,25].

Thus, in order to face the global spread of anxiety and depression among youth as a consequence of the pandemic [4], emotional intelligence must be promoted, cultivated and integrated in educational settings [33]. In the current opinion paper, we will address the interconnectedness between motion, emotion and cognition (see Figure 1), and their importance for healthy development, especially during this changing post-pandemic era. We will underline the importance of a holistic and integrated view on development and education, focusing not only on academic achievements and cognitive functions, but also on emotional and physical health and well-being through an increased experience of the embodied self. We will now look at this in light of the Sphere Model of Consciousness (SMC; Refs. [34,35,36,37]).

## 2. Sphere Model of Consciousness, Emotional Intelligence, Electrophysiological and Neuroanatomical Data

**Figure 2:**
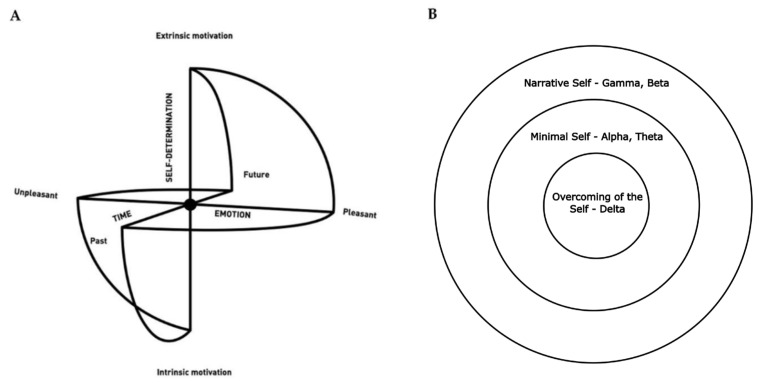
The Sphere Model of Consciousness (**A**) The SMC is configured into three main axes: (1) time, (2) emotion and (3) self-determination, which represent the features of human experience [24,34,35,36,37,38] (**B**) and its three types of Self: Narrative Self (outermost circle), Minimal Self (middle circle) and Overcoming of the Self (innermost circle) with the associated prevalent frequency bands, with slower bands as we go towards the center of the sphere. Adapted from: [38].

SMC is a neurodevelopmental model of consciousness which highlights the relevance of self-determination as a driving force enabling change [34,35,36,37], which can be applied to the motion/emotion/cognition triangle in the context of learning [26] and in the case of motor control, regulation of emotion and cognition. The SMC represents also three different types of Self as concentric circles around a center (Figure 2). The Narrative Self (NS), on the outermost layer, which relates to autobiographical memories, projections into the future, conceptual contents and continuous awareness of personal identity, is the most common type we experience. The Minimal Self (MS) relates to the awareness of the body as a sensorimotor unit, the embodied selfhood anchored in the “here and now”. Finally, the Overcoming of the Self (OtS) represents a state in which any sense of self disappears.

Moving toward the center of the sphere means moving from the more projective dimension of the Narrative Self, related mostly to default mode network activity [39,40], to Minimal Self, in which we are more connected to the present through the body. Being present to oneself is a necessary condition for enhancing awareness of emotions and the ability to regulate them [41]. The SMC can, thus, have an inherent educative element, as it provides the student with visual feedback with respect to an inner state/position and, eventually, a way to move from it toward the center [35,36,37]. Becoming more aware of oneself is crucial to develop emotional intelligence and reach emotional well-being [41].

Several studies and meta-analyses of school-based stress-prevention programming emphasize the effectiveness of addressing social and emotional variables to enhance positive youth development and mental health and educational outcomes [42,43,44]. However, being emotionally intelligent without being self-aware will not bring the desired results. For example, as Gohm and coworkers [45] have previously highlighted, people who had high emotional intelligence potential but reported low scores of awareness of their level of emotional intelligence experienced more stress compared to people with higher scores of self-awareness [45]. In other words, to develop and improve emotional skills you must have self-awareness [46].

In this regard, the SMC, which has been recognized by UNESCO as a useful tool in innovative educational methods [47], emphasizes embodied cognition [35,38,48], the central role of an integrated perspective on Self and the importance of self-awareness. In previous studies on SMC, the focus was on the effects of cortical and cerebellar synchronization on enhancing cognitive functions [49]. Yet these are equally important also for emotional well-being. Studies on physical activity have already indicated that more effective improvement in executive functioning resulted from an approach combining cognitive, physical, and emotional engagement, compared to relying on a single component alone [50,51], demonstrating the importance of addressing jointly the wider spectrum of functioning. The learning process requires the aforementioned executive functioning in the form of attention and memory, but it is also facilitated by a relaxed state [52]. In this context, it is important to note that this state can be achieved through movement meditation, such as the Quadrato Motor Training (QMT), a specific form of movement meditation [49,53]. In the next section (Section 3), we will highlight recent electrophysiological and molecular results addressing the aforementioned domains, reporting recent studies that employed the QMT among healthy and non-healthy participants. The QMT can foster this state as an efficient way of distancing oneself from the NS and strengthening the connection to the body and internally directed attention [54].

A recent review [38] addressed the SMC and its concentric organization of Self according to present results in electrophysiological literature. In this review, authors highlighted how each type of Self, and the cognitive, emotional and bodily functions associated to them, are related and display predominant activity in specific frequency bands [38]. In particular, a crucial position in the SMC is occupied by Alpha (8–12 Hz) and Theta (4–7 Hz) frequency bands, due to their important involvement in the embodied awareness and for their association with the MS and its central position in the SMC between NS and OtS. Alpha and Theta frequencies increase in more relaxed states, lower arousal, are associated with meditation and have an internally directed focus of attention [55,56,57,58], all related to MS. In addition, both Alpha and Theta are closely related to executive functions, such as working memory [59]. Alpha further plays a crucial role in cortical inhibition [60,61] and cognitive flexibility [62,63]. Of particular importance in the search for awareness and its electrophysiological correlates, Sugimura and colleagues [64] showed that participants who tested well in having a strong sense of who they are (i.e., identity synthesis) exhibited increased frontal Theta. In addition, frontocentral Alpha negatively correlated with identity confusion [64]. Alpha activity is further known to increase following different sensorimotor trainings [65]. These results suggest how a stronger sense of oneself is reported in MS, which is characterized by bodily perception and a more consistent and continuous self-perception compared to NS and linked to Alpha and Theta frequency bands.

Thus, Alpha and Theta and MS are strongly linked with the integration between bodily and cognitive domains, but, according to SMC (Figure 2) and the model presented above (Figure 1), we are still missing one critical element for a holistic view: the affective domain. Evidence shows that subjective emotional experience is significantly correlated with Theta, whereas internalized attention with Theta and Alpha decrease synchronization, highlighting important associations of Theta and Alpha in oscillating network activity with states of internalized attention and positive emotional experience [66] (for a review on the neurophysiology of meditation see [57]). Instead, specifically with regard to Alpha, results from different studies have found a connection between Alpha activity and emotion regulation. For example, individuals with high emotional intelligence, defined as the ability to recognize emotion and utilize emotions and emotion-related information as part of general problem-solving [67], compared with participants with average emotional intelligence, displayed decreased upper Alpha event-related desynchronization (ERD) in tasks related to emotion recognition and processing [68,69]. Furthermore, Choi and colleagues [70] showed that relative left frontal activity increased while using a reappraisal strategy for negative images compared to normally viewing same images [70]. Another recent study [71] highlighted how frontal Alpha asymmetry during resting state could predict emotion regulation difficulties, reporting that participants with higher relative left frontal activity have less difficulties in emotion regulation, especially in the dimension of impulse control [71].

The role of Alpha is closely related also to cerebellar functioning. It has been proposed that cerebellar Alpha oscillatory activity may mediate both cortical and cerebellar communication [72,73]. This is further evidenced by different studies that have highlighted that sensorimotor cerebellar stimulation may modulate neural activity in frontal regions, crucial for cognitive functions, such as planning and creativity [62,74,75]. As cerebellar lesions have effects beyond motor functions and cognitive processes, interesting evidence in this regard is the existence of the Cerebellar Cognitive Affective Syndrome (CCAS). Schmahmann and Sherman [76] showed a consistent pattern of deficits and impairments associated with cerebellar lesions: disturbances in both “cool” executive functions (e.g., deficits in planning, set-shifting, abstract reasoning, working memory and verbal fluency) and “hot” executive functions, such as emotion-related changes (e.g., flattening or blunting of affect, disinhibited or inappropriate behavior) [76]. The importance of cerebellar oscillatory activity has also been acknowledged in neuroplasticity [77,78] and, more interestingly, studies have already demonstrated cerebellar microstructural changes following a sensorimotor training [79,80]. In particular, we propose that paradigms such as the QMT could benefit the integrated development of students through both the faster electrophysiological path, and the longer neuroanatomical one, mediated by molecular change. Its focus on cerebellar involvement, neurobiological factors and the junction between functional and structural pathways makes Ben-Soussan and colleagues’ model [49] a novel way to conceptualize the integration of motion, emotion and cognitive development and could help in creating a new method of policymaking regarding the importance of movement in schools and assigning more movement-based hours in the curriculum, that can, in turn, help in dealing with anxiety and depression by going beyond the NS to the MS, or, as Adele Diamond in 2010 wrote: “If we want the best academic outcomes, the most efficient and cost-effective route to achieve that is, counterintuitively, not to narrowly focus on academics, but to also address children’s social, emotional, and physical development” [81]. Moreover, finding ways to optimally engage students in the learning process and outcomes should be a primary concern for teachers and educators [82].

In the next section, we will examine more in detail the Quadrato Motor Training (QMT) and its potentiality in improving cognitive and emotional aspects in school and non-school settings, addressing, in an integrated way, the different domains of functioning of participants across different fields of research.

## 3. Quadrato Motor Training and Its Two Paths of Effects

**Figure 3:**
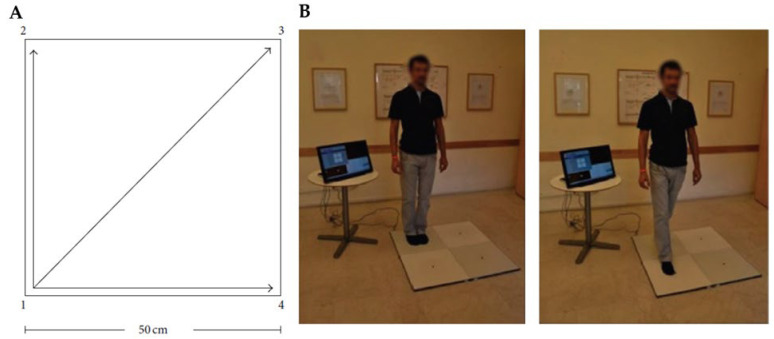
Quadrato Motor Training (QMT). (**A**) A graphical illustration of QMT. (**B**) A participant waiting for the next QMT instruction (**left**) and following the QMT instruction (**right**).

Quadrato Motor Training (QMT) is a non-aerobic cognitively engaging movement practice which requires coordination, working memory and divided attention [83,84], in which the participant has to alternate between movements and staying still, while focusing on their bodies in the present moment and excluding every possible distraction. QMT is conducted on a 50 × 50 cm square, known as the Quadrato space. Its corners are labelled with the numbers 1–4. Participants are required to either produce or inhibit a motor response in the Quadrato space on the basis of specific verbal instructions presented. Motor responses consist of steps in one of three possible directions: right or left; forward or backward; or diagonally (e.g., a verbal instruction can be “1–2”, which directs the practitioner to take a step forward from corner number 1 to corner number 2). When two numbers of the verbal instruction are the same (e.g., “2–2”), participants must inhibit the impulse to move upon hearing the voice command and wait for the next instruction. This inhibitory control (cognitive and motor) required to make a decision based on the specific verbal instruction and not automatically is one of the main features of the QMT. Inhibition is also involved in keeping their eyes focused forward with their hands by their sides, and in following the next instruction without stopping, even in case of errors (Figure 3) [53,84]. QMT, thus, requires executive functions, such as motor coordination, balance, awareness of the body and its location in space, together with cognitive elaboration and error monitoring, as well as regulation of behavior involving emotional content [84]. In other words, it involves functions related to the cognitive, emotional and motor dimensions.

Effects of QMT have been studied in different contexts, age groups and samples, demonstrating how QMT is able to improve cognitive and psycho-emotional functioning [25,54,83,85,86,87]. More specifically, QMT has been found to increase ideational flexibility, an index of creativity and divergent thinking, in both adults [83] and school-aged children [54], as well as to enhance self-efficacy and affect balance [83]. Furthermore, evidence suggests that QMT is strongly linked to internally oriented attention and to increased reflectivity, mindfulness and altered states of consciousness [85,88].

From an electro-physiological perspective, different studies on healthy participants showed how QMT increases Alpha power and coherence in the [83,85,89,90]. Specifically, Ben-Soussan and colleagues [83] demonstrated that improvements in ideational flexibility were concurrent with enhanced intra- and inter-hemispheric synchronization. Increased Alpha coherence was mainly found in bilateral fronto-temporal networks and frontal areas and was also confirmed in a later study using resting state electroencephalography (rsEEG) [90]. It is important to highlight in this regard that increased hemispheric Alpha has been found to be linked to enhanced cognitive functions due to a better integration of information and communication among different brain areas [91,92]. Interestingly, QMT-elicited changes in neural activity, such as frontal Alpha and contingent negative variation amplitude [83,93], are known to be closely related to planning and decision making [94,95], fundamental skills in life and especially for students and adolescents. In addition, increased Alpha power is related to an internally directed focus of attention [55,56,58,96] and to increased creativity, as well as to the experience of flow [62,97]. Flow, which can be described as a state in which one is so interested and pleasurably involved in an activity (e.g., painting, reading, playing, etc.) that everything else is in the background [98], is particularly interesting from an educational perspective because it addresses the aforementioned need to involve students in first person in the process of learning.

Thus, current available electrophysiological literature on the QMT shows that executing specifically structured movements can lead to integrated communication between brain areas associated with cognition and emotion and, due to its connection with divergent thinking, creativity and flow, the QMT may be a very important tool to employ in school settings and with school-aged children, as it was already proven to be in recent years [54].

Notwithstanding interesting electrophysiological results, in order to better support the usefulness of the QMT in educational settings, it is also crucial to address the corresponding changes in the brain and cerebellum from a neuroanatomical perspective. Evidence shows that QMT increases cerebellum and frontal lobe grey matter [49,86], and that this increase is coupled with higher white matter integrity in the corpus callosum, anterior thalamic radiations, corticospinal tracts, cerebellar peduncles, uncinate fasciculi and superior longitudinal fasciculi [86,87,99]. These results are quite significant for healthy development and neuroeducational programs, as white matter increase in the anterior thalamic radiations is generally related to executive functions such as memory and behavior planning [100,101]. Moreover, the increase in limbic-frontal connectivity is often involved in adaptive behavioral responses, self-efficacy and emotional balance [84]. Finally, enhanced white matter integrity in the superior longitudinal fascicule has been positively associated with increased ideational flexibility [86] and general self-efficacy [87].

In addition to electrophysiological and anatomical effects, recent studies demonstrated that QMT also induces molecular changes [99,102,103,104]. In particular, results showed how, after 4 weeks of QMT practice, participants showed decreased salivary proNGF (neurotrophine precursor of nerve growth factor), correlated with increased ideational flexibility and creativity in both adults and children [103]. Other results reported increased salivary proBDNF (neurotrophine precursor of brain-derived neurotrophic factor) after 12 weeks of QMT practice [99]. Finally, another recent case-study with a severely dyslexic individual showed that QMT elicited an increase in proNGF levels after 4 weeks and an increase in proBDNF levels after 10 weeks, confirming the patterns of a “early” effect in proNGF and a “late” increase in proBDNF with regard to QMT practice [104].

How are these changes in neurotrophine precursor levels related to harmonic development and how can they impact the lives and well-being of students? Firstly, proBDNF and NGF have been found to be linked to learning, spatial cognition and neuronal plasticity [105,106,107,108]. Secondly, NGF, aside from its neurodevelopmental importance and its crucial role in regulating the survival, growth and differentiation of neurons in the peripheral and central nervous systems, which have been studied for the last 70 years [109,110,111,112,113], also has sensitizing effects on nociceptors that result in hyperalgesia [114]. Finally, NGF-dependent neurons mediate the reciprocal communication between the brain and immune and endocrine systems [115] and contribute to the processes of interoception, inflammation, homeostasis and the response to stress [116].

Taken together, evidence shows how QMT elicits changes across functional, structural and molecular fields (see Table 1 for a summary of experimental studies on QMT).

**Figure 4:**
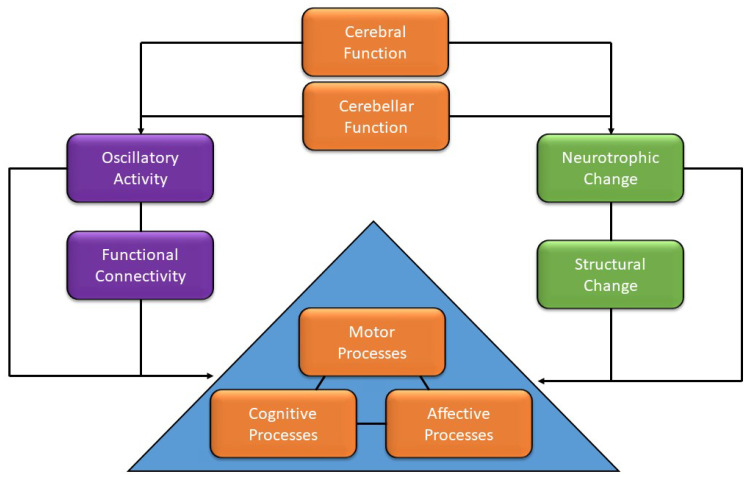
Interconnected relationship between cerebellar and cognitive, motor and affective functions. The relationship is mediated via two routes. The first involves oscillatory activity through functional changes in connectivity. The second involves molecular effects through structural changes in connectivity.

Moreover, aforementioned changes affect a wide spectrum of different domains, such as executive functions and affective processes, both crucial in everyday life and especially important for students. Thus, techniques such as the QMT may be capable of fostering a harmonic development for participants, through the dual functional and structural pathways highlighted in Figure 4.

## 4. Discussion

Recently, Stodden and colleagues [26] raised the need for a new and more integrated view to conceptualize school and, in general, the learning processes. This concern has been previously raised by many different researchers, who highlighted that not only cognitive processes are at play during learning [25,81,123,124,125,126,127]. Nonetheless, an integrated view of conventional education comprising of both bodily, cognitive and emotional aspects has yet to be reached. A new way to operate schools and, in a sense, to teach and learn, is especially important in current post-pandemic times, when, more than ever, children, adolescents and even adults are under enormous levels of stress and their mental health is at risk [2,3]. These high levels of stress are not only a concern for the well-being of the population but also for the functioning of individuals and society. Even low levels of stress can severely and negatively impact the prefrontal cortex [128] and, by extension, executive functions and cognitive flexibility. Conversely, reducing stress in schools leads to decreased teacher burn-out and to better academic outcomes [129,130,131,132], because children feel safer and able to fully explore the possibilities of learning without worrying. Motivation and engagement are also dependent upon whether the child or adolescent feels safe, physically and psychologically [133]. Consequently, positive social and motivational climate characteristics are crucial for ensuring motivation and emotional well-being in students [133,134,135,136,137].

Thus, education and learning are not only cognitive, but often rely and depend on affective components, such as emotional intelligence and motivation. On the other hand, our cognitive and affective functioning is also significantly increased by physical fitness, with the prefrontal cortex and executive functions being the greatest beneficiaries [138,139]. One possible reason for this is that cognitive and motor functions rely approximately on same or substantially overlapping brain regions [140,141]. This holds true even for psychopathology, evidence shows that sensory and motor secondary symptoms may indeed be important indicators of underlying network aberrations across a wide range of psychopathologies [142]. An intertwined relationship between different domains is present also in mental disorders, such as attention deficit hyperactivity disorder (ADHD) and autism spectrum disorders (ASD), or in immune dysregulation, with people with a high IQ being more likely to being diagnosed compared to national average statistics, supporting the idea of a hyper brain/hyper body association (i.e., hyper brain = high IQ, hyper body = elevated sensory, and altered immune and inflammatory responses; [143]).

An important issue remains to be solved: how much of this integrated and holistic perspective can be implemented in a normal/conventional school curriculum?

In spite of evidence suggesting that children should engage in physical activity for at least 60 min every day [144], children are prevalently sedentary and do not meet physical activity guidelines [145,146]. Given that children spend most of their time in school, a feasible and effective way to reduce sitting time and sedentary behavior is performing classroom-based activities, such as active breaks [147]. Active breaks requiring cognitive engagement (e.g., trying to toss a light ball to each other in class without talking or dropping the ball) are indeed effective at reducing sitting times and improving executive functions, but the implementation of these activities might require some time for teachers and schools to be effectively employed [16].

Another possible way to improve the emotional well-being of students and reduce stress is practicing contemplative practices in school. A review of 15 school meditation programs showed how meditation was beneficial in the majority of considered studies with regard to well-being, social competence and academic achievement [148]. Results also highlighted how school meditation programs were more effective if the practice occurred daily and was delivered by the teacher [148]. Notwithstanding the good efficacy of this type of program, by having the teacher delivering the practice and given that the majority of meditation types lack physical activity involvement, this might result in a difficult implementation/not complete school-based activity.

Conversely, we highlighted how, an embodied and sensorimotor training, the Quadrato Motor Training has demonstrated the capacity to elicit concrete functional, structural and molecular changes not only on the dyad motion-cognition but also reintegrating the emotional aspect in this often-dominating duality, jointly addressing aforementioned crucial factors for the learning process. In addition, QMT is a relatively easy and short training (around 7 min), with simple and practicable movements that can be practiced anywhere, given that it requires very little space, and, after a few practice sessions with a specialized trainer, can be performed in total autonomy. Importantly, QMT has already been tested, with good preliminary results in a school setting [54] and, together with other practices discussed above, can constitute a good answer to the needs of students of all ages, especially in these volatile times.

Notwithstanding positive changes highlighted in the present opinion paper, the effectiveness of this integrated approach needs to be thoroughly examined with several well-structured, well-powered experimental paradigms, addressing bodily, cognitive and emotional variables with multiple appropriate measures assessing the feasibility of possible interventions.

## 5. Conclusions

Implementing mindful movements practices [149], such as the QMT, by bringing focused awareness and concentration to experience movement of the body in the here-and-now can have many advantages. Moreover, contemplative practice with a specific attention to the body and bodily awareness, such as Vipassana meditation and One Minute Meditation (OMM; [150]), may also elicit similar emotional effects in a school setting. Thus, future studies should compare QMT to additional embodied movements and contemplative practices, utilizing a large cohort of students in different age groups and comparing the effectiveness in a school setting in reducing stress and improving affective, cognitive and motor processes.

Educational programs often rely on cognitive factors, sometimes in combination with physical and affective involvement, but very rarely will programs involve all three domains in attempting to improve students’ learning and wellbeing. We suggested how this partial involvement may not be the optimal way to preserve health, and therefore learning, in the post pandemic era. For this reason, we highlighted how QMT could be a cost-effective and easy-to-learn possibility to address, in an integrated way, motion, emotion and cognition. Operating on an integrated level could allow teachers and educators to improve the learning process, jointly with fostering students’ resilience and protection to stress, thus promoting physical and mental health with the student at the center of this action, helping children and adolescents to learn and adapt better to the ever-changing reality we live in.

## Figures and Tables

**Table 1:** Summary of the experimental studies on QMT [54,83,85,85,87,88,90,93,99,102,103,104,117,118,119,120,121,122].

Study	Participants	Design	Duration	Measures	Outcomes
Ben Soussan et al., 2013	N = 27; 20–35 years *	Between groups comparison: QMT; Verbal Training; Simple Motor Training	Single training	Pre-post evaluation: EEG; Reaction Time Task; AUT	Enhanced inter-hemispheric and intra-hemispheric alpha coherence; increased divergent thinking (AUT) scores pre-post in QMT group compared to both control groups
Ben-Soussan et al., 2014a	N = 22; 2 groups of 10 healthy and 12 dyslexic participants	Longitudinal	4 weeks of daily training	Pre-post evaluation: MEG; Reading Test; Category-based Fluency Task; Letter-based Fluency Task	Increased cerebellar alpha power after QMT in dyslexic participants; Improved performance on Reading Test after QMT in both dyslexic and healthy participants
Ben-Soussan et al., 2014b (Study A)	N = 24; 3 groups of 9, 7 and 8 participants	Between groups comparison: QMT; Verbal Training; Simple Motor Training	Single training	Pre-post evaluation: HFT	Improved performance on spatial cognition (HFT) in QMT group compared to two control groups
Ben-Soussan et al., 2014b (Study B)	N = 37	Longitudinal	Single training	Pre-post evaluation: EEG; HFT	Enhanced theta and alpha intra-hemispheric coherence in females; Reduced theta and alpha intra-hemispheric coherence in males; spatial cognition (HFT) performance improved in both genders after QMT;
Ben-Soussan et al., 2014c	N = 3; 29, 49 and 53 years	Longitudinal	12 weeks of daily training	Pre-post evaluation: MRI; Salivary proBDNF	ProBDNF levels increased after QMT; GM volume increased bilaterally in the cerebellum, in right thalamus and limbic lobe; WMI increased in the corpus callosum, anterior thalamic radiations, corticospinal tracts, cerebellar peduncles, and superior longitudinal fascicule; ProBDNF levels positively correlated with GM and WMI
Venditti et al., 2015	N = 40; 2 groups of 20 participants	Between groups comparison: QMT; Simple Motor Training	4 weeks of daily training	Pre-post evaluation: Salivary proNGF; AUT	Increased divergent thinking (AUT) scores in the QMT group; Change in scores negatively correlated with the change in proNGF levels in QMT group.
Ben-Soussan et al., 2015	N = 27; 20–35 years *	Between groups comparison: QMT; Verbal Training; Simple Motor Training	4 weeks of daily training	Pre-post evaluation: MRI; AUT	GM volume and WMI changes in cerebellum, inferior frontal and middle frontal gyri; Increased divergent thinking (AUT) scores in the QMT group compared to control groups; Anatomical changes were positively correlated with cognitive flexibility scores
Ben-Soussan et al., 2017	N = 43; 3 groups of 15, 14 and 14 participants	Between groups comparison: BM practitioners (1 week of QMT); BM practitioners (4 weeks of QMT); controls (4 weeks of QMT)	4 weeks of daily training	Pre-post evaluation: First-person reports	Increased reports of attention, mindfulness, ability to wait, positive emotions, bodily harmony, spontaneous visualization, sense of wonder
Lasaponara et al., 2017	N = 50; 25–45 years *	Longitudinal	12 weeks of daily training	Evaluation in T0-T1-T2: EEG	Increased limbic and fronto-temporal alpha connectivity after 6 weeks of QMT; Increased occipital alpha connectivity after 12 weeks of QMt
Paoletti et al., 2017	N = 84; 2 groups of 42 participants	Between groups comparison: BM with/without QMT	7 days of daily training	Pre-post evaluation: ABS; GSE	Higher increase in affective balance (ABS) score for the QMT group compared to control group
Piervincenzi et al., 2017	N = 50; 25–45 years *	Longitudinal	12 weeks of daily training	Evaluation in T0-T1-T2: MRI; AUT; GSE; The Motivation Scale	Bilateral increase in WMI after 6 weeks of QMT (T1) in tracts related to sensorimotor and cognitive functions; WMI increments still present after 12 weeks of QMT (T2) in the left hemisphere; Significant correlations between WMI changes and increased scores in self-efficacy (GSE) and divergent thinking (AUT)
Ben-Soussan and Glicksohn, 2018	N = 29; 3 groups of 9, 10 and 10 participants	Between groups comparison: Healthy-Verbal Training; Healthy-QMT; Dyslexic-QMT	4 weeks of daily training	Pre-post evaluation: TP Task	Compared to Verbal training group, longer TP after QMT in the in dyslexic females; Shorter TP after QMT in healthy females
Verdone et al., 2018	N = 1; 20 years old male dyslexic participant	Longitudinal	10 weeks of daily training	Pre-post evaluation: Salivary proBDNF and proNGF; Reading Test; AUT	Increased salivary proBDNF and proNGF after QMT; Improved performance on Reading Test after QMT; Increased divergent thinking (AUT) scores after QMT
Ben-Soussan et al., 2019	N = 34; 3 groups of 11, 9 and 14 participants	Between groups comparison: Aikido practitioners; QMT practitioners; passive control group	Observational	TP; Homolateral interlimb coordination Task	Longer and more accurate time duration estimations in QMT group compared to Aikido and passive control groups
Caserta et al., 2019	N = 40; 2 groups of 20 participants	Between groups comparison: QMT; Simple Motor Training	12 weeks of daily training	Pre-post evaluation: Salivary proBDNF and proNGF	proNGF level increased in QMT group while proBDNF showed no significant change. No correlation between the two neurotrophins prior to training was detectable, but a significant positive correlation between change in proNGF and proBDNF after training
Lasaponara et al., 2019	N = 23; 19–41 years	Longitudinal	Single training	Pre-post evaluation: EEG; SRT; CRT	CNV amplitude (change in alertedness) reduced in CRT and increased in SRT after QMT; P3 amplitude (cognitive load and novelty detection) increased in CRT and decreased in SRT after QMT
Ben-Soussan et al., 2020	N = 50; 25–45 years *	Longitudinal	6 weeks of daily training	Pre-post evaluation: MRI; First-person reports	Silence experience positively correlated with longitudinal WMI increments in the left Uncinate Fasciculus; First-person reports of experiencing silence and reduced mind-wandering
Marson et al., 2021	N = 50; 4 class from 5th to 8th grade	All participants (crossover design) performed: QMT; OMM	5 weeks of daily training	Pre-post evaluation: HFT; AUT	Younger children showed increased spatial cognition (HFT) and divergent thinking (AUT) scores after QMT; Older children showed increased divergent thinking (AUT) scores after OMM and increased spatial cognition (HFT) after both trainings
Verdone et al., 2023	N = 30; 2 groups of 15 participants	Between groups comparison: QMT; passive control group	8 weeks of daily training	Pre-post evaluation: Salivary IL-1β; HFT; AUT	pro-inflammatory (IL-1β protein) level decreased and AUT scores in QMT group increased compared to the control group

* Same sample. **CNV** = Contingent Negative Variation; **WMI** = White Matter Integrity; **GM** = Gray Matter; **BDNF**= Brain-Derived Neutotrophic Factor; **NGF** = Nerve Growth Factor; **AUT** = Alternative Uses Task; **HFT** = Hidden Figures Test; **ABS** = Affect Balance Scale; **GSE** = General Self-Efficacy Scale; **TP** = Time Production; **SRT** = Simple Reaction Time Task; **CRT** = Choice Reaction Time Task; **OMM** = One Minute Meditation; **BM** = Breathing Meditation.

## Data Availability

Data sharing not applicable.

## References

[1] GBD 2019 Mental Disorders Collaborators (2022). Global, Regional, and National Burden of 12 Mental Disorders in 204 Countries and Territories, 1990–2019: A Systematic Analysis for the Global Burden of Disease Study 2019. Lancet Psychiatry.

[2] Santomauro D.F., Herrera A.M.M., Shadid J., Zheng P., Ashbaugh C., Pigott D.M., Abbafati C., Adolph C., Amlag J.O., Aravkin A.Y. (2021). Global Prevalence and Burden of Depressive and Anxiety Disorders in 204 Countries and Territories in 2020 Due to the COVID-19 Pandemic. Lancet.

[3] Yao H., Chen J.-H., Xu Y.-F. (2020). Patients with Mental Health Disorders in the COVID-19 Epidemic. Lancet Psychiatry.

[4] Racine N., McArthur B.A., Cooke J.E., Eirich R., Zhu J., Madigan S. (2021). Global Prevalence of Depressive and Anxiety Symptoms in Children and Adolescents during COVID-19: A Meta-Analysis. JAMA Pediatr..

[5] UNESCO (2021). Education: From Disruption to Recovery.

[6] Ebert D.D., Mortier P., Kaehlke F., Bruffaerts R., Baumeister H., Auerbach R.P., Alonso J., Vilagut G., Martínez K.U., Lochner C. (2019). Barriers of Mental Health Treatment Utilization among First-year College Students: First Cross-national Results from the WHO World Mental Health International College Student Initiative. Int. J. Methods Psychiatr. Res..

[7] Benjet C. (2020). Stress Management Interventions for College Students in the Context of the COVID-19 Pandemic. Clin. Psychol. Sci. Pract..

[8] Amanvermez Y., Rahmadiana M., Karyotaki E., de Wit L., Ebert D.D., Kessler R.C., Cuijpers P. (2020). Stress Management Interventions for College Students: A Systematic Review and Meta-Analysis. Clin. Psychol. Sci. Pract..

[9] De Meester A., Stodden D., Goodway J., True L., Brian A., Ferkel R., Haerens L. (2018). Identifying a Motor Proficiency Barrier for Meeting Physical Activity Guidelines in Children. J. Sci. Med. Sport.

[10] Opstoel K., Chapelle L., Prins F.J., De Meester A., Haerens L., van Tartwijk J., De Martelaer K. (2020). Personal and Social Development in Physical Education and Sports: A Review Study. Eur. Phys. Educ. Rev..

[11] Tait J.L., Collyer T.A., Gall S.L., Magnussen C.G., Venn A.J., Dwyer T., Fraser B.J., Moran C., Srikanth V.K., Callisaya M.L. (2022). Longitudinal Associations of Childhood Fitness and Obesity Profiles with Midlife Cognitive Function: An Australian Cohort Study. J. Sci. Med. Sport.

[12] Van der Fels I.M., Te Wierike S.C., Hartman E., Elferink-Gemser M.T., Smith J., Visscher C. (2015). The Relationship between Motor Skills and Cognitive Skills in 4–16 Year Old Typically Developing Children: A Systematic Review. J. Sci. Med. Sport.

[13] Katzmarzyk P.T., Denstel K.D., Beals K., Carlson J., Crouter S.E., McKenzie T.L., Pate R.R., Sisson S.B., Staiano A.E., Stanish H. (2018). Results from the United States 2018 Report Card on Physical Activity for Children and Youth. J. Phys. Act. Health.

[14] Chen T.J., Watson K.B., Michael S.L., Carlson S.A. (2021). Sex-Stratified Trends in Meeting Physical Activity Guidelines, Participating in Sports, and Attending Physical Education among US Adolescents, Youth Risk Behavior Survey 2009–2019. J. Phys. Act. Health.

[15] Brian A., Pennell A., Taunton S., Starrett A., Howard-Shaughnessy C., Goodway J.D., Wadsworth D., Rudisill M., Stodden D. (2019). Motor Competence Levels and Developmental Delay in Early Childhood: A Multicenter Cross-Sectional Study Conducted in the USA. Sports Med..

[16] Mazzoli E., Salmon J., Teo W.-P., Pesce C., He J., Ben-Soussan T.D., Barnett L.M. (2021). Breaking up Classroom Sitting Time with Cognitively Engaging Physical Activity: Behavioural and Brain Responses. PLoS ONE.

[17] Lees C., Hopkins J. (2013). Peer Reviewed: Effect of Aerobic Exercise on Cognition, Academic Achievement, and Psychosocial Function in Children: A Systematic Review of Randomized Control Trials. Prev. Chronic. Dis..

[18] Best J.R., Miller P.H. (2010). A Developmental Perspective on Executive Function. Child Dev..

[19] Robbins S.B., Lauver K., Le H., Davis D., Langley R., Carlstrom A. (2004). Do Psychosocial and Study Skill Factors Predict College Outcomes? A Meta-Analysis. Psychol. Bull..

[20] Sanchez-Ruiz M.-J., Mavroveli S., Poullis J. (2013). Trait Emotional Intelligence and Its Links to University Performance: An Examination. Personal. Individ. Differ..

[21] Sanchez-Ruiz M.-J., El Khoury J., Saadé G., Salkhanian M. (2016). Non-Cognitive Variables and Academic Achievement: The Role of General and Academic Self-Efficacy and Trait Emotional Intelligence. Non-Cognitive Skills and Factors in Educational Attainment.

[22] Leisman G., Moustafa A.A., Shafir T. (2016). Thinking, Walking, Talking: Integratory Motor and Cognitive Brain Function. Front. Public Health.

[23] Leisman G., Machado C., Melillo R., Mualem R. (2012). Intentionality and “Free-Will” from a Neurodevelopmental Perspective. Front. Integr. Neurosci..

[24] Paoletti P. (2008). Crescere Nell’eccellenza.

[25] Pesce C., Ben-Soussan T.D. (2016). “Cogito Ergo Sum” or “Ambulo Ergo Sum”? New Perspectives in Developmental Exercise and Cognition Research. Exercise-Cognition Interaction: Neuroscience Perspectives.

[26] Stodden D.F., Pesce C., Zarrett N., Tomporowski P., Ben-Soussan T.D., Brian A., Abrams T., Weist M.D. (2023). Holistic Functioning from a Developmental Perspective: A New Synthesis with a Focus on a Multi-Tiered System Support Structure. Clin. Child Fam. Psychol. Rev..

[27] Soto-Rubio A., Giménez-Espert M.d.C., Prado-Gascó V. (2020). Effect of Emotional Intelligence and Psychosocial Risks on Burnout, Job Satisfaction, and Nurses’ Health during the Covid-19 Pandemic. Int. J. Environ. Res. Public. Health.

[28] Goleman D. (1998). Working with Emotional Intelligence.

[29] Lea R.G., Davis S.K., Mahoney B., Qualter P. (2019). Does Emotional Intelligence Buffer the Effects of Acute Stress? A Systematic Review. Front. Psychol..

[30] Pool L.D., Qualter P. (2018). An Introduction to Emotional Intelligence.

[31] Schneider T.R., Lyons J.B., Khazon S. (2013). Emotional Intelligence and Resilience. Personal. Individ. Differ..

[32] Luthar S.S., Cicchetti D., Becker B. (2000). Research on Resilience: Response to Commentaries. Child Dev..

[33] Paoletti P., Di Giuseppe T., Lillo C., Ben-Soussan T.D., Bozkurt A., Tabibnia G., Kelmendi K., Warthe G.W., Leshem R., Bigo V. (2022). What Can We Learn from the COVID-19 Pandemic? Resilience for the Future and Neuropsychopedagogical Insights. Front. Psychol..

[34] Paoletti P. (2002). Flussi, Territori, Luogo [Flows, Territories, Place].

[35] Paoletti P., Ben Soussan T.D. (2019). The Sphere Model of Consciousness: From Geometrical to Neuro-Psycho-Educational Perspectives. Log. Univers..

[36] Paoletti P., Ben-Soussan T.D. (2020). Reflections on Inner and Outer Silence and Consciousness without Contents According to the Sphere Model of Consciousness. Front. Psychol..

[37] Paoletti P., Ben-Soussan T.D., Glicksohn J. (2020). Inner Navigation and Theta Activity: From Movement to Cognition and Hypnosis According to the Sphere Model of Consciousness. Hypnotheraphy and Hypnosis.

[38] Paoletti P., Leshem R., Pellegrino M., Ben-Soussan T.D. (2022). Tackling the Electro-Topography of the Selves through the Sphere Model of Consciousness. Front. Psychol..

[39] Davey C.G., Harrison B.J. (2018). The Brain’s Center of Gravity: How the Default Mode Network Helps Us to Understand the Self. World Psychiatry.

[40] Mehl-Madrona L., Mainguy B. (2022). Neuroscience and Narrative. Anthropol. Conscious..

[41] Paoletti P., Ben-Soussan T.D. (2021). Emotional Intelligence, Identification, and Self-Awareness According to the Sphere Model of Consciousness. Sci. Emot. Intell..

[42] Catalano R.F., Berglund M.L., Ryan J.A., Lonczak H.S., Hawkins J.D. (2002). Positive Youth Development in the United States: Research Findings on Evaluations of Positive Youth Development Programs. Prev. Treat..

[43] Durlak J.A., Weissberg R.P. (2007). The Impact of After-School Programs That Promote Personal and Social Skills. Collab. Acad. Soc. Emot. Learn. NJ1.

[44] Greenberg M.T., Weissberg R.P., O’Brien M.U., Zins J.E., Fredericks L., Resnik H., Elias M.J. (2003). Enhancing School-Based Prevention and Youth Development through Coordinated Social, Emotional, and Academic Learning. Am. Psychol..

[45] Gohm C.L., Corser G.C., Dalsky D.J. (2005). Emotional Intelligence under Stress: Useful, Unnecessary, or Irrelevant?. Personal. Individ. Differ..

[46] Drigas A., Papoutsi C. (2020). The Need for Emotional Intelligence Training Education in Critical and Stressful Situations: The Case of Covid-19. Int. J. Recent Contrib. Eng. Sci. IT.

[47] Colonna A. (2020). Creating Communities of Knowledge and Connecting to Landscape. Humanistic Futures of Learning: Perspectives from UNESCO Chairs and UNITWI N Networks.

[48] Pintimalli A., Glicksohn J., Marson F., Di Giuseppe T., Ben-Soussan T.D. (2023). Change in Time Perception Following the Place of Pre-Existence Technique. Int. J. Environ. Res. Public. Health.

[49] Ben-Soussan T.D., Glicksohn J., Berkovich-Ohana A. (2015). From Cerebellar Activation and Connectivity to Cognition: A Review of the Quadrato Motor Training. BioMed Res. Int..

[50] Pesce C. (2012). Shifting the Focus from Quantitative to Qualitative Exercise Characteristics in Exercise and Cognition Research. J. Sport Exerc. Psychol..

[51] Tomporowski P.D., Pesce C. (2019). Exercise, Sports, and Performance Arts Benefit Cognition via a Common Process. Psychol. Bull..

[52] Huggins E., Ennis M., Zuttermeister P. (2000). Academic Performance among Middle School Students after Exposure to a Relaxation Response Curriculum. J. Res. Dev. Educ..

[53] De Fano A., Leshem R., Ben-Soussan T.D. (2019). Creating an Internal Environment of Cognitive and Psycho-Emotional Well-Being through an External Movement-Based Environment: An Overview of Quadrato Motor Training. Int. J. Environ. Res. Public Health.

[54] Marson F., Fano A.D., Pellegrino M., Pesce C., Glicksohn J., Ben-Soussan T.D. (2021). Age-Related Differential Effects of School-Based Sitting and Movement Meditation on Creativity and Spatial Cognition: A Pilot Study. Children.

[55] Cooper N.R., Croft R.J., Dominey S.J., Burgess A.P., Gruzelier J.H. (2003). Paradox Lost? Exploring the Role of Alpha Oscillations during Externally vs. Internally Directed Attention and the Implications for Idling and Inhibition Hypotheses. Int. J. Psychophysiol..

[56] Takahashi T., Murata T., Hamada T., Omori M., Kosaka H., Kikuchi M., Yoshida H., Wada Y. (2005). Changes in EEG and Autonomic Nervous Activity during Meditation and Their Association with Personality Traits. Int. J. Psychophysiol..

[57] Lomas T., Ivtzan I., Fu C.H. (2015). A Systematic Review of the Neurophysiology of Mindfulness on EEG Oscillations. Neurosci. Biobehav. Rev..

[58] Cona G., Chiossi F., Di Tomasso S., Pellegrino G., Piccione F., Bisiacchi P., Arcara G. (2020). Theta and Alpha Oscillations as Signatures of Internal and External Attention to Delayed Intentions: A Magnetoencephalography (MEG) Study. NeuroImage.

[59] Sauseng P., Klimesch W., Doppelmayr M., Pecherstorfer T., Freunberger R., Hanslmayr S. (2005). EEG Alpha Synchronization and Functional Coupling during Top-down Processing in a Working Memory Task. Hum. Brain Mapp..

[60] Klimesch W., Sauseng P., Hanslmayr S. (2007). EEG Alpha Oscillations: The Inhibition–Timing Hypothesis. Brain Res. Rev..

[61] Klimesch W. (2011). Evoked Alpha and Early Access to the Knowledge System: The P1 Inhibition Timing Hypothesis. Brain Res..

[62] Fink A., Graif B., Neubauer A.C. (2009). Brain Correlates Underlying Creative Thinking: EEG Alpha Activity in Professional vs. Novice Dancers. NeuroImage.

[63] Wolff N., Zink N., Stock A.-K., Beste C. (2017). On the Relevance of the Alpha Frequency Oscillation’s Small-World Network Architecture for Cognitive Flexibility. Sci. Rep..

[64] Sugimura K., Iwasa Y., Kobayashi R., Honda T., Hashimoto J., Kashihara S., Zhu J., Yamamoto K., Kawahara T., Anno M. (2021). Association between Long-Range Temporal Correlations in Intrinsic EEG Activity and Subjective Sense of Identity. Sci. Rep..

[65] Kerr C.E., Sacchet M.D., Lazar S.W., Moore C.I., Jones S.R. (2013). Mindfulness Starts with the Body: Somatosensory Attention and Top-down Modulation of Cortical Alpha Rhythms in Mindfulness Meditation. Front. Hum. Neurosci..

[66] Aftanas L.I., Golocheikine S.A. (2001). Human Anterior and Frontal Midline Theta and Lower Alpha Reflect Emotionally Positive State and Internalized Attention: High-Resolution EEG Investigation of Meditation. Neurosci. Lett..

[67] Mayer J.D., Caruso D.R., Salovey P. (1999). Emotional Intelligence Meets Traditional Standards for an Intelligence. Intelligence.

[68] Jaušovec N., Jaušovec K. (2005). Differences in Induced Gamma and Upper Alpha Oscillations in the Human Brain Related to Verbal/Performance and Emotional Intelligence. Int. J. Psychophysiol..

[69] Lee J.Y., Lindquist K.A., Nam C.S. (2017). Emotional Granularity Effects on Event-Related Brain Potentials during Affective Picture Processing. Front. Hum. Neurosci..

[70] Choi D., Sekiya T., Minote N., Watanuki S. (2016). Relative Left Frontal Activity in Reappraisal and Suppression of Negative Emotion: Evidence from Frontal Alpha Asymmetry (FAA). Int. J. Psychophysiol..

[71] Zhang J., Hua Y., Xiu L., Oei T.P., Hu P. (2020). Resting State Frontal Alpha Asymmetry Predicts Emotion Regulation Difficulties in Impulse Control. Personal. Individ. Differ..

[72] Kujala J., Pammer K., Cornelissen P., Roebroeck A., Formisano E., Salmelin R. (2007). Phase Coupling in a Cerebro-Cerebellar Network at 8–13 Hz during Reading. Cereb. Cortex.

[73] Silberstein R.B., Danieli F., Nunez P.L. (2003). Fronto-Parietal Evoked Potential Synchronization Is Increased during Mental Rotation. NeuroReport.

[74] Dietrich A. (2006). Transient Hypofrontality as a Mechanism for the Psychological Effects of Exercise. Psychiatry Res..

[75] Watson T.C., Becker N., Apps R., Jones M.W. (2014). Back to Front: Cerebellar Connections and Interactions with the Prefrontal Cortex. Front. Syst. Neurosci..

[76] Schmahmann J.D., Sherman J.C. (1998). The Cerebellar Cognitive Affective Syndrome. Brain J. Neurol..

[77] Swinnen S.P. (2002). Intermanual Coordination: From Behavioural Principles to Neural-Network Interactions. Nat. Rev. Neurosci..

[78] De Zeeuw C.I., Hoebeek F.E., Bosman L.W., Schonewille M., Witter L., Koekkoek S.K. (2011). Spatiotemporal Firing Patterns in the Cerebellum. Nat. Rev. Neurosci..

[79] Carbon M., Kingsley P.B., Tang C., Bressman S., Eidelberg D. (2008). Microstructural White Matter Changes in Primary Torsion Dystonia. Mov. Disord..

[80] Carbon M., Argyelan M., Habeck C., Ghilardi M.F., Fitzpatrick T., Dhawan V., Pourfar M., Bressman S.B., Eidelberg D. (2010). Increased Sensorimotor Network Activity in DYT1 Dystonia: A Functional Imaging Study. Brain.

[81] Diamond A. (2010). The Evidence Base for Improving School Outcomes by Addressing the Whole Child and by Addressing Skills and Attitudes, Not Just Content. Early Educ. Dev..

[82] Shernoff D.J., Csikszentmihalyi M., Shneider B., Shernoff E.S. (2003). Student Engagement in High School Classrooms from the Perspective of Flow Theory. Sch. Psychol. Q..

[83] Dotan Ben-Soussan T., Glicksohn J., Goldstein A., Berkovich-Ohana A., Donchin O. (2013). Into the Square and out of the Box: The Effects of Quadrato Motor Training on Creativity and Alpha Coherence. PLoS ONE.

[84] Leshem R., De Fano A., Ben-Soussan T.D. (2020). The Implications of Motor and Cognitive Inhibition for Hot and Cool Executive Functions: The Case of Quadrato Motor Training. Front. Psychol..

[85] Ben-Soussan T.D., Avirame K., Glicksohn J., Goldstein A., Harpaz Y., Ben-Shachar M. (2014). Changes in Cerebellar Activity and Inter-Hemispheric Coherence Accompany Improved Reading Performance Following Quadrato Motor Training. Front. Syst. Neurosci..

[86] Ben-Soussan T.D., Berkovich-Ohana A., Piervincenzi C., Glicksohn J., Carducci F. (2015). Embodied Cognitive Flexibility and Neuroplasticity Following Quadrato Motor Training. Front. Psychol..

[87] Piervincenzi C., Ben-Soussan T.D., Mauro F., Mallio C.A., Errante Y., Quattrocchi C.C., Carducci F. (2017). White Matter Microstructural Changes Following Quadrato Motor Training: A Longitudinal Study. Front. Hum. Neurosci..

[88] Ben-Soussan T.D., Glicksohn J., Berkovich-Ohana A. (2017). Attentional Effort, Mindfulness, and Altered States of Consciousness Experiences Following Quadrato Motor Training. Mindfulness.

[89] Stefano L., Federica M., Ben-Soussan T.D., Filippo C., Tombini M., Quattrocchi C.C., Yuri E., Mallio C.A., Patrizio P. (2016). Electrophysiological Indexes of Eyes Open and Closed Resting States Conditions Following the Quadrato Motor Training. Int. J. Bioelectromagn..

[90] Lasaponara S., Mauro F., Carducci F., Paoletti P., Tombini M., Quattrocchi C.C., Mallio C.A., Errante Y., Scarciolla L., Ben-Soussan T.D. (2017). Increased Alpha Band Functional Connectivity Following the Quadrato Motor Training: A Longitudinal Study. Front. Hum. Neurosci..

[91] Sauseng P., Klimesch W., Stadler W., Schabus M., Doppelmayr M., Hanslmayr S., Gruber W.R., Birbaumer N. (2005). A Shift of Visual Spatial Attention Is Selectively Associated with Human EEG Alpha Activity. Eur. J. Neurosci..

[92] Engel T.A., Steinmetz N.A., Gieselmann M.A., Thiele A., Moore T., Boahen K. (2016). Selective Modulation of Cortical State during Spatial Attention. Science.

[93] Lasaponara S., Glicksohn J., Mauro F., Ben-Soussan T.D. (2019). Contingent Negative Variation and P3 Modulations Following Mindful Movement Training. Prog. Brain Res..

[94] Harung H., Travis F., Blank W., Heaton D. (2009). Higher Development, Brain Integration, and Excellence in Leadership. Manag. Decis..

[95] Travis F., Harung H.S., Lagrosen Y. (2011). Moral Development, Executive Functioning, Peak Experiences and Brain Patterns in Professional and Amateur Classical Musicians: Interpreted in Light of a Unified Theory of Performance. Conscious. Cogn..

[96] Shaw J.C. (1996). Intention as a Component of the Alpha-Rhythm Response to Mental Activity. Int. J. Psychophysiol..

[97] Katahira K., Yamazaki Y., Yamaoka C., Ozaki H., Nakagawa S., Nagata N. (2018). EEG Correlates of the Flow State: A Combination of Increased Frontal Theta and Moderate Frontocentral Alpha Rhythm in the Mental Arithmetic Task. Front. Psychol..

[98] Csikszentmihalyi M., Larson R. (2014). Flow and the Foundations of Positive Psychology.

[99] Ben-Soussan T.D., Piervincenzi C., Venditti S., Verdone L., Caserta M., Carducci F. (2015). Increased Cerebellar Volume and BDNF Level Following Quadrato Motor Training. Synapse.

[100] Mamah D., Conturo T.E., Harms M.P., Akbudak E., Wang L., McMichael A.R., Gado M.H., Barch D.M., Csernansky J.G. (2010). Anterior Thalamic Radiation Integrity in Schizophrenia: A Diffusion-Tensor Imaging Study. Psychiatry Res. Neuroimaging.

[101] Van Der Werf Y.D., Jolles J., Witter M.P., Uylings H.B. (2003). Contributions of Thalamic Nuclei to Declarative Memory Functioning. Cortex.

[102] Caserta M., Ben-Soussan T.D., Vetriani V., Venditti S., Verdone L. (2019). Influence of Quadrato Motor Training on Salivary ProNGF and ProBDNF. Front. Neurosci..

[103] Venditti S., Verdone L., Pesce C., Tocci N., Caserta M., Ben-Soussan T.D. (2015). Creating Well-Being: Increased Creativity and ProNGF Decrease Following Quadrato Motor Training. BioMed Res. Int..

[104] Verdone L., Caserta M., Vetriani V., Glicksohn J., Venditti S., Ben-Soussan T.D. (2019). Molecular and Cognitive Effects of Quadrato Motor Training in Adult Dyslexia: A Longitudinal Case Study. Brain Body Cogn..

[105] Mandel R., Gage F., Thal L. (1989). Spatial Learning in Rats: Correlation with Cortical Choline Acetyltransferase and Improvement with NGF Following NBM Damage. Exp. Neurol..

[106] Angelucci F., De Bartolo P., Gelfo F., Foti F., Cutuli D., Bossù P., Caltagirone C., Petrosini L. (2009). Increased Concentrations of Nerve Growth Factor and Brain-Derived Neurotrophic Factor in the Rat Cerebellum after Exposure to Environmental Enrichment. Cerebellum.

[107] Conner J.M., Franks K.M., Titterness A.K., Russell K., Merrill D.A., Christie B.R., Sejnowski T.J., Tuszynski M.H. (2009). NGF Is Essential for Hippocampal Plasticity and Learning. J. Neurosci..

[108] Fritsch B., Reis J., Martinowich K., Schambra H.M., Ji Y., Cohen L.G., Lu B. (2010). Direct Current Stimulation Promotes BDNF-Dependent Synaptic Plasticity: Potential Implications for Motor Learning. Neuron.

[109] Levi-Montalcini R., Hamburger V. (1951). Selective Growth Stimulating Effects of Mouse Sarcoma on the Sensory and Sympathetic Nervous System of the Chick Embryo. J. Exp. Zool..

[110] Levi-Montalcini R. (1952). Effects of Mouse Tumor Transplantation on the Nervous System. Ann. N. Y. Acad. Sci..

[111] Verge V., Richardson P., Wiesenfeld-Hallin Z., Hokfelt T. (1995). Differential Influence of Nerve Growth Factor on Neuropeptide Expression in Vivo: A Novel Role in Peptide Suppression in Adult Sensory Neurons. J. Neurosci..

[112] Han Z., Wang C.-P., Cong N., Gu Y.-Y., Ma R., Chi F.-L. (2017). Therapeutic Value of Nerve Growth Factor in Promoting Neural Stem Cell Survival and Differentiation and Protecting against Neuronal Hearing Loss. Mol. Cell. Biochem..

[113] Schwarz S., Lehmbecker A., Tongtako W., Hahn K., Wang Y., Felmy F., Zdora I., Brogden G., Branitzki-Heinemann K., von Köckritz-Blickwede M. (2020). Neurotrophic Effects of GM1 Ganglioside, NGF, and FGF2 on Canine Dorsal Root Ganglia Neurons in Vitro. Sci. Rep..

[114] Denk F., Bennett D.L., McMahon S.B. (2017). Nerve Growth Factor and Pain Mechanisms. Annu. Rev. Neurosci..

[115] Indo Y. (2012). Nerve Growth Factor and the Physiology of Pain: Lessons from Congenital Insensitivity to Pain with Anhidrosis. Clin. Genet..

[116] Indo Y. (2018). NGF-Dependent Neurons and Neurobiology of Emotions and Feelings: Lessons from Congenital Insensitivity to Pain with Anhidrosis. Neurosci. Biobehav. Rev..

[117] Ben-Soussan T.D., Berkovich-Ohana A., Glicksohn J., Goldstein A. (2014). A Suspended Act: Increased Reflectivity and Gender-Dependent Electrophysiological Change Following Quadrato Motor Training. Front. Psychol..

[118] Paoletti P., Glicksohn J., Ben-Soussan T.D. (2017). Inner Design Technology: Improved Affect by Quadrato Motor Training. The Amygdala—Where Emotions Shape Perception, Learning and Memories.

[119] Ben-Soussan T.D., Glicksohn J. (2018). Gender-Dependent Changes in Time Production Following Quadrato Motor Training in Dyslexic and Normal Readers. Front. Comput. Neurosci..

[120] Ben-Soussan T.D., Glicksohn J., De Fano A., Mauro F., Marson F., Modica M., Pesce C. (2019). Embodied Time: Time Production in Advanced Quadrato and Aikido Practitioners. PsyCh J..

[121] Ben-Soussan T.D., Marson F., Piervincenzi C., Glicksohn J., De Fano A., Amenduni F., Quattrocchi C.C., Carducci F. (2020). Correlates of Silence: Enhanced Microstructural Changes in the Uncinate Fasciculus. Front. Psychol..

[122] Verdone L., Marson F., Caserta M., Zampieri M., Reale A., Bacalini M.G., Vetriani V., Ben-Soussan T.D., Venditti S. (2023). Quadrato Motor Training (QMT) Influences IL-1β Expression and Creativity: Implications for Inflammatory State Reduction and Cognitive Enhancement. Progress in Brain Research.

[123] Rosenthal R., Jacobsen L. (1968). Pygmalion in the Classroom: Self-Fulfilling Prophecies and Teacher Expectations.

[124] Flink C., Boggiano A.K., Barrett M. (1990). Controlling Teaching Strategies: Undermining Children’s Self-Determination and Performance. J. Pers. Soc. Psychol..

[125] Flink C., Boggiano A.K., Main D.S., Barrett M., Katz P. (1992). Children’s Achievement-Related Behaviors: The Role of Extrinsic and Intrinsic Motivational Orientations. Achievement and Motivation: A Social-Developmental Perspective.

[126] Steele C.M., Aronson J. (1995). Attitudes and Social Cognition. J. Pers. Soc. Psychol..

[127] Aronson J., Cohen G., Nail P.R. (1999). Self-Affirmation Theory: An Update and Appraisal. Cognitive Dissonance: Reexamining a Pivotal Theory in Psychology.

[128] Arnsten A.F. (1998). Catecholamine Modulation of Prefrontal Cortical Cognitive Function. Trends Cogn. Sci..

[129] Jethwani-Keyser M.M. (2008). “When Teachers Treat Me Well, I Think I Belong”: School Belonging and the Psychological and Academic Well Being of Adolescent Girls in India. Ph.D. Thesis.

[130] Jennings P.A., Greenberg M.T. (2009). The Prosocial Classroom: Teacher Social and Emotional Competence in Relation to Student and Classroom Outcomes. Rev. Educ. Res..

[131] Denham S.A., Brown C. (2010). “Plays Nice with Others”: Social–Emotional Learning and Academic Success. Early Educ. Dev..

[132] Downer J., Sabol T.J., Hamre B. (2010). Teacher–Child Interactions in the Classroom: Toward a Theory of within-and Cross-Domain Links to Children’s Developmental Outcomes. Early Educ. Dev..

[133] National Research Council (2002). Community Programs to Promote Youth Development.

[134] Shernoff D.J. (2010). Engagement in After-School Programs as a Predictor of Social Competence and Academic Performance. Am. J. Community Psychol..

[135] Dawes N.P., Larson R. (2011). How Youth Get Engaged: Grounded-Theory Research on Motivational Development in Organized Youth Programs. Dev. Psychol..

[136] Zarrett N., Sorensen C., Cook B.S. (2015). Physical and Social-Motivational Contextual Correlates of Youth Physical Activity in Underresourced Afterschool Programs. Health Educ. Behav..

[137] Zarrett N., Abraczinskas M., Skiles Cook B., Wilson D.K., Ragaban F. (2018). Promoting Physical Activity within Under-Resourced Afterschool Programs: A Qualitative Investigation of Staff Experiences and Motivational Strategies for Engaging Youth. Appl. Dev. Sci..

[138] Hillman C.H., Erickson K.I., Kramer A.F. (2008). Be Smart, Exercise Your Heart: Exercise Effects on Brain and Cognition. Nat. Rev. Neurosci..

[139] Hillman C.H., Buck S.M., Themanson J.R., Pontifex M.B., Castelli D.M. (2009). Aerobic Fitness and Cognitive Development: Event-Related Brain Potential and Task Performance Indices of Executive Control in Preadolescent Children. Dev. Psychol..

[140] Diamond A. (2000). Close Interrelation of Motor Development and Cognitive Development and of the Cerebellum and Prefrontal Cortex. Child Dev..

[141] Rosenbaum D.A., Carlson R.A., Gilmore R.O. (2001). Acquisition of Intellectual and Perceptual-Motor Skills. Annu. Rev. Psychol..

[142] Levit-Binnun N., Davidovitch M., Golland Y. (2013). Sensory and Motor Secondary Symptoms as Indicators of Brain Vulnerability. J. Neurodev. Disord..

[143] Karpinski R.I., Kolb A.M.K., Tetreault N.A., Borowski T.B. (2018). High Intelligence: A Risk Factor for Psychological and Physiological Overexcitabilities. Intelligence.

[144] World Health Organization (2010). Global Recommendations on Physical Activity for Health.

[145] Colley R.C., Garriguet D., Janssen I., Craig C.L., Clarke J., Tremblay M.S. (2011). Physical Activity of Canadian Children and Youth: Accelerometer Results from the 2007 to 2009 Canadian Health Measures Survey. Health Rep..

[146] Cliff D.P., Hesketh K.D., Vella S.A., Hinkley T., Tsiros M.D., Ridgers N.D., Carver A., Veitch J., Parrish A., Hardy L.L. (2016). Objectively Measured Sedentary Behaviour and Health and Development in Children and Adolescents: Systematic Review and Meta-analysis. Obes. Rev..

[147] Watson A., Timperio A., Brown H., Best K., Hesketh K.D. (2017). Effect of Classroom-Based Physical Activity Interventions on Academic and Physical Activity Outcomes: A Systematic Review and Meta-Analysis. Int. J. Behav. Nutr. Phys. Act..

[148] Waters L., Barsky A., Ridd A., Allen K. (2015). Contemplative Education: A Systematic, Evidence-Based Review of the Effect of Meditation Interventions in Schools. Educ. Psychol. Rev..

[149] Russell T.A., Arcuri S.M. (2015). A Neurophysiological and Neuropsychological Consideration of Mindful Movement: Clinical and Research Implications. Front. Hum. Neurosci..

[150] Paoletti P. (2018). OMM: The One Minute Meditation.

